# COVID-19 Aftermath: Exploring the Mental Health Emergency among Students at a Northern Italian University

**DOI:** 10.3390/ijerph19148587

**Published:** 2022-07-14

**Authors:** Alessandra Patrono, Stefano Renzetti, Angela Manco, Paola Brunelli, Stefanny M. Moncada, Mark J. Macgowan, Donatella Placidi, Stefano Calza, Giuseppa Cagna, Matteo Rota, Maurizio Memo, Maurizio Tira, Roberto G. Lucchini

**Affiliations:** 1 Department of Molecular and Translational Medicine, University of Brescia, 25121 Brescia, Italy; stefano.calza@unibs.it (S.C.); matteo.rota@unibs.it (M.R.); maurizio.memo@unibs.it (M.M.); 2 Department of Medical-Surgical Specialties, Radiological Sciences and Public Health, University of Brescia, 25121 Brescia, Italy; stefano.renzetti@unibs.it (S.R.); a.manco@studenti.unibs.it (A.M.); p.brunelli002@studenti.unibs.it (P.B.); donatella.placidi@unibs.it (D.P.); giuseppa.cagna@unibs.it (G.C.); rlucchin@fiu.edu (R.G.L.); 3 Departamento de Gobierno, Universidad del Desarrollo, Santiago 7590000, Chile; stefanny.moncada@gmail.com; 4 School of Social Work, Robert Stempel College of Public Health and Social Work, Florida International University, Miami, FL 33199, USA; macgowan@fiu.edu; 5 Department of Civil, Environmental, Architectural Engineering and Mathematics (DICATAM), University of Brescia, 25121 Brescia, Italy; maurizio.tira@unibs.it; 6 Department of Environmental Health Sciences, School of Public Health, Florida International University, Miami, FL 33199, USA

**Keywords:** mental health, psychosomatic effect, young adults, COVID-19

## Abstract

In this study, we investigated the symptoms of physical and mental health associated with lifestyle changes due to a lockdown among the students of a university in Northern Italy, one of the most affected areas in Europe during the first wave of COVID-19. We examined the psychopathological variations in relation to mental health problems in a young population. The goal was to develop interventions to resolve these new psychosocial problems. From June to July 2020, students participated in an anonymous survey asking about habits and symptoms that emerged during the lockdown and the COVID-19 pandemic. Five health outcomes were assessed: digestive disorders; headaches; fear of COVID-19; panic and anxiety crises; and depression/sadness. The conditions and duration of the social isolation, lifestyle, SARS-CoV-2 infection in the household, financial situation, and productivity were considered in the analysis. A total of 3533 students completed the survey. The participants experienced headaches, depression and sadness, digestive disorders, a fear of COVID-19, and anxiety/panic crises. The duration of isolation was associated with an increased risk of digestive disorders, headaches, and COVID-19 fear. The female gender, medium–intense telephone usage, sleep quality, memory difficulties, and performance reduction were associated with an increased risk of the health outcomes. Future interventions should focus on promoting and implementing different habits with the support of health and university organizations.

## 1. Introduction

Coronavirus disease (COVID-19) has shown various health consequences, including cardiovascular and respiratory failure leading to death [1]. Uncertainty over the course of the pandemic combined with health implications and lifestyle changes has increased psychological distress worldwide [2]. Social isolation due to restrictive measures during the COVID-19 pandemic has caused serious challenges with potential, and unprecedented, long-term consequences. Higher levels of perceived stress [3] and psychological distress [4] have been experienced.

At the beginning of March 2020, the Italian government established rigorous forms of containment to limit the spread of the virus, including closing schools and universities as well as non-essential goods stores and places of social gathering. University students reported greater loneliness and less time spent studying together with other colleagues compared with pre-pandemic life [5]. Social isolation, a fear of a SARS-CoV-2 infection, and socio-economic concerns related to the pandemic have been shown to significantly impact the young adult population [6], especially among women [7,8], the unemployed, and individuals with a lower socio-economic status [9,10]. Young adults are particularly susceptible to the emergence of mental illness [11] and the incidence of anxiety and depression among young adults increased during the pandemic [12].

A cross-sectional survey in Brazil identified young adults, women, and people with a history of depression as being at a high risk of developing sadness, frequent nervousness, and sleep disorders associated with the pandemic [13]. Other longitudinal and large-scale population studies have shown that acute stress, anxiety, and depressive symptoms were prevalent among college students during the COVID-19 outbreak with a significant increase after the initial phase of the outbreak [14]. The pandemic had psychological effects in several ways. In the literature, there is evidence that people have been particularly vulnerable to the emergence of problems of a psychosomatic nature due to COVID-19 [15]. The possibility that stressful events can lead to physical symptoms of a psychological nature has been widely documented [16,17]. Considering how physical symptoms can be a reflection of psychological distress [18], we decided to evaluate the presence of these symptoms in a population of young adults.

The COVID-19 pandemic increased mental health difficulties among university students who showed depression, anxiety, suicidal thoughts, and academic concerns with alarming trends given the prolonged experience of social isolation [18]. Therefore, universities should provide additional support to students to face mental health problems [19].

During the first wave of the COVID-19 pandemic, the most affected area in Italy was the southeast part of the Lombardy region. The University of Brescia is the main public university in this area and one of the largest in Northern Italy. Loneliness and social isolation are risk factors for depression, anxiety, chronic stress, insomnia, and dementia in old age [20]; therefore, a study focusing on the impact of forced social isolation in young adults is highly relevant. The aim of this study was to investigate the psychological and somatic symptoms driven by lifestyle changes due to the pandemic restrictions among the students of the University of Brescia. The results can help to identify strategies and interventions that can be utilized by universities to improve the well-being of students and prevent university dropout situations.

## 2. Materials and Methods

### 2.1. Study Population

All students (15,261) from the University of Brescia enrolled at the School of Medicine, Engineering, Economics, and Law were asked to participate through the institutional mailing list. Overall, 3533 students (2773 Bachelor’s degree students, 518 Master’s degree students, and 242 doctoral students and residents) answered the questionnaire, resulting in an overall participation rate of 23.2%. The Ethics Committee of the Province of Brescia granted an exemption from its authorization as the data collected were anonymous.

### 2.2. Enrollment and Questionnaire

The survey was designed to identify the potential negative implications of social isolation and to identify protective factors to draw preventive recommendations to avoid health impacts among the students. All answers of the students were collected through the Google Forms database. We focused this survey on the isolation period related to the first national lockdown in Italy, which occurred from 9 March 2020 to 3 May 2020; the survey remained open for 49 consecutive days starting from 3 June 2020 to 22 July 2020. The self-report questionnaire was prepared by a working group that included occupational doctors and psychologists. The investigators balanced trying to use as few items as possible to make it easy to administer with collecting an adequate amount of information. There were 74 questions and it took about 10 to 15 min to complete.

The questionnaire was structured into ten sections: (1) socio-demographic information, including gender, age, and nationality; (2) academic data, such as the educational qualification and the disciplinary area of studies; (3) degree course, in reference to which degree the student was enrolled in; (4) socio-economic status (SES), either as an individual or family and categorized into low, medium, and high; (5) conditions of social isolation, including information on housing conditions and access to a private garden, hours spent away from home, and the use of screen time (time spent in front of the television, video games or a phone) expressed in hours. The frequency of use was then explored as increased, decreased, or unchanged compared with the pre-pandemic period; (6) lifestyle during the lockdown, including questions on alcohol consumption, cigarette smoking, and eating habits. Sleep quality was assessed through a frequency scale, indicating any changes if increased, decreased, or unchanged. Changes in dietary habits were assessed by investigating the prevalence of the consumption of food categories (e.g., fish and carbohydrates) compared with pre-isolation habits; (7) physical and mental health symptoms, including a fear of infection. A few items were inspired by the Adult Behavior Checklist (ABCL) [21] for the construction of the questionnaire: the relevance of dealing with topics such as physical health symptoms (digestive problems, headaches, joint pain, and sleep disturbances) considered the psychosomatic symptoms in association with other symptoms such as anxiety states and depression. The presence of physical symptoms was assessed by asking the participant to respond to the presence or absence of symptoms with a “yes/no” answer. The perception of anxiety and depression was rated on a 5-point Likert scale from “never” to “often”; (8) SARS-CoV-2 infection in the household, assessed through questions on having symptoms or being positive for COVID-19 as well as information on the death of family members due to COVID-19; (9) economic and financial situation, to assess potential concerns about the possibility of continue studying; and (10) productivity, assessed as a perception of learning and concentration difficulties.

### 2.3. Statistical Analysis

The descriptive analysis consisted of reporting the median values as well as the first and third quartiles to describe the continuous variables because of their skewed distribution. Absolute and relative frequencies were applied to the categorical variables. The impact of social isolation on physical and mental health was assessed considering five different outcomes: digestive disorders; headaches; a fear of COVID-19; anxiety or panic; and sadness/depression.

The inferential data analysis was conducted considering the influence of various covariates: the housing conditions during lockdown; the possibility of leaving the house during the day; and the lifestyles of the participants as potential protective or harmful factors. An exploratory factor analysis was used to establish whether the 27 variables in the questionnaire could be explained by a smaller number of latent variables. As all variables were dichotomous or ordinal, we applied a polychoric correlation matrix. The suitability of the data for the exploratory factor analysis was assessed through Bartlett’s test of sphericity and the Kaiser–Meyer–Olkin (KMO) criterion. A label was attributed to each factor based on the loadings associated with each original variable: the threshold for factor loadings was set to 0.3 for inclusion in a factor. Logistic and ordinal logistic regression models (depending on whether the dependent variable was dichotomous or with more ordinal categories, respectively) were then considered to evaluate the impact of the latent variables defined through the factor analysis as well as the socio-demographic data (age, sex, and SES) and the duration of social isolation. All model estimates were obtained through a maximum likelihood estimation. A statistical significance level was assumed at *p* < 0.05. The data were analyzed through the statistical program R (v. 4.1.0).

## 3. Results

### 3.1. Descriptive Statistics

A total of 3533 students (23.2% of the enrolled students at UNIBS) participated in the survey with a higher participation rate among medical students (39.3%). Most of the respondents were females (59.0%) with a median age of 22 years (range 18–40), mainly with a medium SES level (77.8%) and residing in the province of Brescia (75.7%) (Table 1 and Table S1). Most responses (99.69%) were received in June 2020, within the first two weeks from the beginning of the survey.

The median duration of the total period of confinement was 63 days; most of the students lived in a house with more than 6 rooms or with a private garden, they shared the same home with 2–3 people, and spent less than an hour away from home on average. The lifestyle questions revealed that most of the target population did not smoke before and during the lockdown (78.5%). Most of those who usually consumed alcohol claimed to have reduced their alcohol consumption (48.1%) and most students increased their time spent undertaking physical exercise (34.3%).

The incidence of symptoms representing the five health outcomes observed during the period of the first lockdown ranged between 13.1% for anxiety/panic crises and 43.6% for headaches (Table 2).

### 3.2. Inferential Data Analysis

#### 3.2.1. Factor Analysis

Bartlett’s test of sphericity (*p* < 0.001) and the KMO criterion (overall KMO value: 0.61) revealed that the data were suitable for an explanatory factor analysis. Only the two variables related to PC usage showed a KMO index below 0.5; however, we decided to include them in the analysis to be able to consider their effect on the outcomes. The factor analysis allowed us to group the 27 variables examined in the questionnaire into the following 11 latent variables (Table 3): TV usage; phone usage; PC usage; video games usage; lockdown conditions; smoking and alcohol habits; unemployed cohabitants during lockdown; having had relatives positive for COVID-19, with symptoms, or deceased; having been positive for or with COVID-19 symptoms; nutrition, weight changes and physical activity; and sleep quality, mnemonic difficulties, and performance reduction.

The coefficient alphas of the internal consistency reliability of the measures ranged between 0.28 and 0.84. The loadings estimated by the factor analysis are shown in the Supplementary Material (Figure S1). The usual sleep quality and lockdown sleep quality showed a negative weight, meaning that a worse usual or lockdown sleep quality implied higher values of the latent variable for sleep quality, mnemonic difficulties, and performance reduction. Similarly, a decrease in weight during lockdown contributed to higher values of the latent variable for nutrition.

#### 3.2.2. Association between Health Outcomes and Covariates

Logistic and ordinal logistic regression analyses showed that age was positively associated with an increased fear of COVID-19 (odds ratio (OR) 1.03; 95% confidence interval (CI) 1.01, 1.05) and negatively associated with headaches and sadness symptoms (OR 0.98; 95% CI 0.96, 1.00 and OR 0.98; 95% CI 0.96, 1.00, respectively). Females showed a higher risk of all symptoms (digestive disorders: OR 2.01; 95% CI 1.65, 2.45; headache: OR 2.52; 95% CI 2.14, 2.98; COVID-19 fear: OR 1.71; 95% CI 1.48, 1.97; anxiety/panic: OR 2.15; 95% CI 1.82, 2.54; sadness/depression: OR 1.89; 95% CI 1.63, 2.18).

A lower SES level was a risk factor for headache symptoms (medium vs. high SES: OR 1.36; 95% CI 1.05, 1.78; low vs. high SES: OR 1.72; 95% CI 1.24, 2.40), anxiety or panic (low vs. high SES: OR 1.44; 95% CI 1.05, 1.99), and sadness or depression (low vs. high SES: OR 1.42; 95% CI 1.06, 1.90).

Considering the latent variables, an increased PC usage resulted in a risk factor for headaches (OR 1.13; 95% CI 1.06, 1.21). Increased phone usage was associated with all health symptoms: sadness/depression (OR 1.20; 95% CI 1.13, 1.27); digestive disorders (OR 1.14; 95% CI 1.05, 1.24); anxiety/panic (OR 1.13; 95% CI 1.06, 1.21); headaches (OR 1.11; 95% CI 1.04, 1.18); and COVID-19 fear (OR 1.11; 95% CI 1.05, 1.17). On the other hand, lockdown conditions where the participants had a private garden, were able to spend hours outside, and had an increased number of cohabitants or rooms in the house significantly decreased the risk of headaches (OR 0.89; 95% CI 0.81, 0.97).

Smoking and alcohol consumption was associated with an increase in digestive disorders (OR 1.20; 95% CI 1.07, 1.35), headaches (OR 1.19; 95% CI 1.08, 1.32), sadness/depression (OR 1.19; 95% CI 1.09, 1.30), and anxiety/panic (OR 1.12; 95% CI 1.02, 1.24). Having unemployed cohabitants during lockdown showed an effect on sadness/depression (OR 1.10; 95% CI 1.03, 1.18). Having had relatives who were COVID-19 positive, with symptoms, or deceased increased the risk of digestive disorders (OR 1.17; 95% CI 1.07, 1.26), anxiety/panic (OR 1.12; 95% CI 1.04, 1.20), and sadness/depression (OR 1.10; 95% CI 1.03, 1.17). Having been positive for COVID-19 or having had COVID-19 symptoms was associated with digestive disorders (OR 1.11; 95% CI 1.05, 1.17).

**Figure 1:**
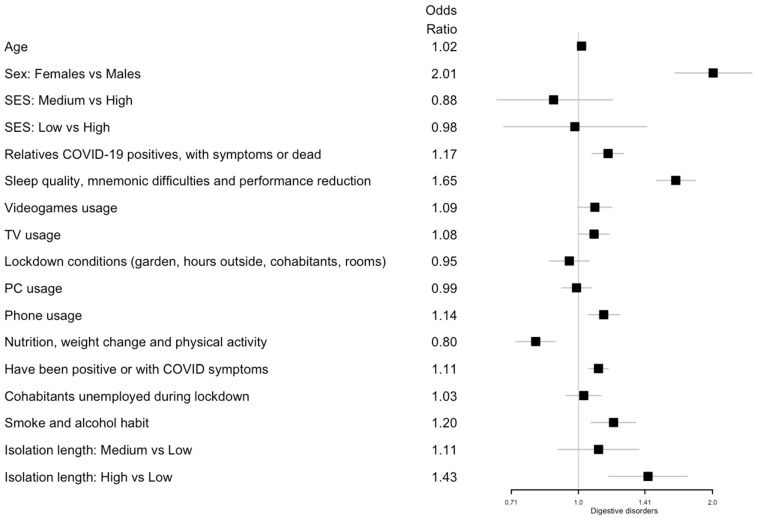
Forest plots for the effect of the covariates on digestive disorders. The squares represent the odds ratios and the lines depict the confidence intervals estimated by the logistic regression.

**Figure 2:**
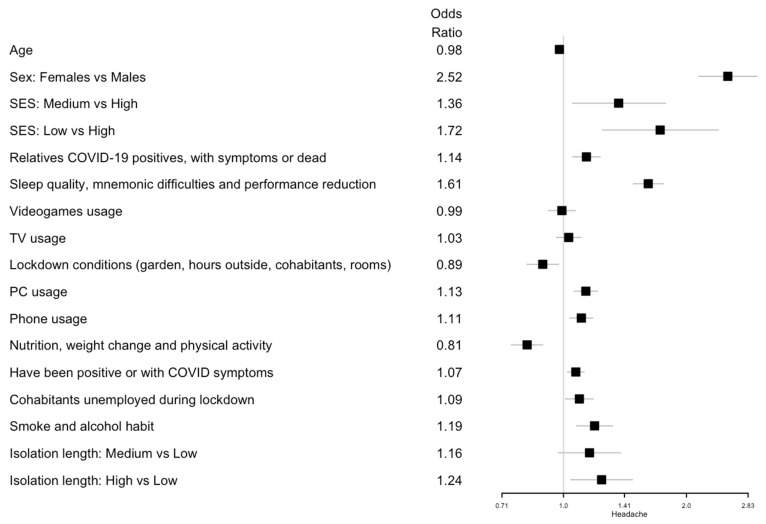
Forest plots for the effect of the covariates on headaches. The squares represent the odds ratios and the lines depict the confidence intervals estimated by the logistic regression.

**Figure 3:**
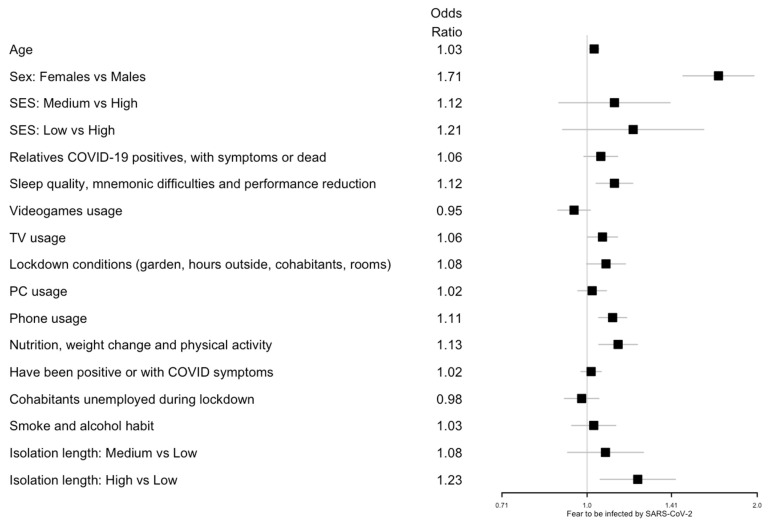
Forest plots for the effect of the covariates on fear of being infected by SARS-CoV-2. The squares represent the odds ratios and the lines depict the confidence intervals estimated by the ordinal logistic regression.

**Figure 4:**
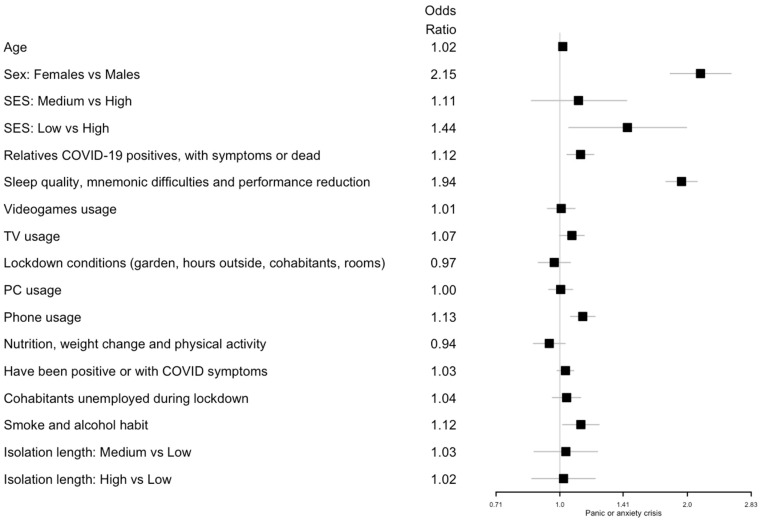
Forest plots for the effect of the covariates on panic or anxiety crises. The squares represent the odds ratios and the lines depict the confidence intervals estimated by the ordinal logistic regression.

**Figure 5:**
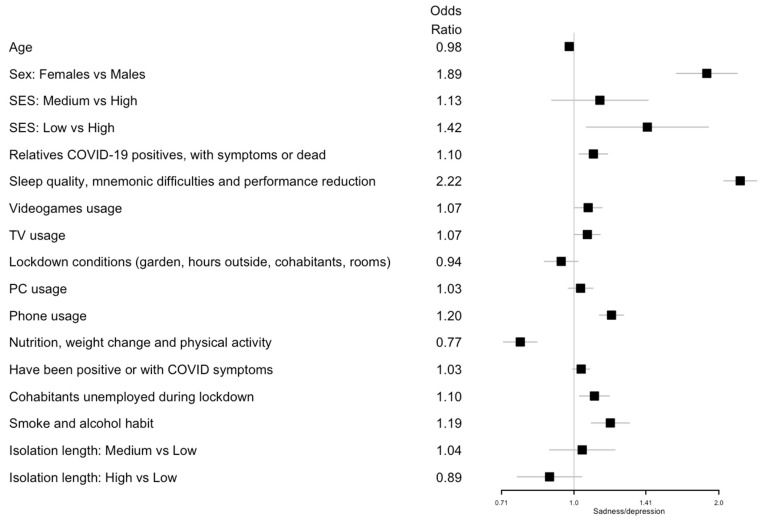
Forest plots for the effect of the covariates on sadness/depression. The squares represent the odds ratios and the lines depict the confidence intervals estimated by the ordinal logistic regression.

Regarding nutrition, weight changes and physical activity as well as an improved diet and physical activity were negatively associated with sadness/depression (OR 0.77; 95% CI 0.71, 0.84), digestive disorders (OR 0.80; 95% CI 0.72, 0.89), and headaches (OR 0.81; 95% CI 0.75, 0.89), but increased the risk of COVID-19 fear (OR 1.13; 95% CI 1.05, 1.23). Poor sleep quality, mnemonic difficulties, and performance reduction were associated with all of the following health symptoms: sadness/depression (OR 2.22; 95% CI 2.05, 2.40); anxiety/panic (OR 1.94; 95% CI 1.78, 2.11); digestive disorders (OR 1.65; 95% CI 1.50, 1.83); headaches (OR 1.61; 95% CI 1.48, 1.76); and COVID-19 fear (OR 1.12; 95% CI 1.04, 1.20). Finally, a long isolation length (more than 71 days) was associated with a higher risk of digestive disorders (OR 1.43; 95% CI 1.17, 1.75), headaches (OR 1.24; 95% CI 1.04, 1.47), and COVID-19 fear (OR 1.23; 95% CI 1.05, 1.43). All the results are summarized in Figure 1 for digestive disorder; Figure 2 for headaches; Figure 3 for fear of being infected by SARS-CoV-2; Figure 4 for panic or anxiety crises; Figure 5 for panic or anxiety crises.

## 4. Discussion

The objective of this survey was to identify particularly relevant risk factors for the onset of psychosomatic symptoms during the COVID-19 pandemic in a significant target population—university students—typically considered at risk of mental health problems [22]. Brescia University is one of the largest in Northern Italy and the province of Brescia was an epicenter of the waves of the pandemic in Italy.

Psychosomatic concerns are an integrative view of health and disease with the interaction between biological, psychological, and social variables [23,24], including the signs and symptoms of a medical disease [25]. Therefore, our overarching aim was to identify and prioritize preventive interventions that can help to prevent or resolve symptoms in population strata particularly vulnerable to the effects of social isolation [26]. The analysis of variations in psychosomatic symptoms among university students should continue to be studied in the current scenario of uncertainty to reduce public health costs and respond to new psychosocial needs.

The results showed that during the lockdown, participants experienced headaches, digestive disorders, a fear of COVID-19, anxiety/panic crises, and depression and sadness. Gender, phone usage, sleep quality, memory difficulties, and performance reduction were associated with all five health outcomes. Habits related to the consumption of alcohol and smoking, having had relatives with COVID-19, or having been positive for COVID-19 or with symptoms as well as an increased time watching TV, a low SES, worsening nutrition, an increased weight, and lower physical activity represented risk factors for the onset of three or four outcomes. These results can contribute to model interventions aimed at preventing or resolving psychosomatic problems in a population particularly vulnerable to the effects of social isolation [26].

A main finding was that females were significantly more susceptible than males in the symptoms considered (digestive disorders, headaches, COVID-19 fear, anxiety/panic, and sadness/depression). This finding was consistent with the existing literature, which shows that women are more prone to anxiety [27,28], depression [29], and a fear of COVID-19 [30]. In addition to genetic and hormonal factors [31], several authors underlined that family tasks and, in a few cases, domestic violence [32] can have a greater impact on the onset of psychosomatic symptoms in women.

Information-communication media (telephones, computers, and televisions) significantly influenced all the health outcomes considered. The pandemic and subsequent social isolation highlighted the previously known relationship between telephone usage and anxiety and depression [33,34]. It is not possible to overlook how televisions, computers, and telephones are a vector of overexposure to information. Social restrictions favored immediate access to screens and the use of television in particular [35], which also exacerbated binge-watching phenomena [36]. The use of screens for entertainment purposes should also be considered due to the significant relationship between playing video games and the symptoms of anxiety/depression. Our research confirmed the findings of a previous study [37] on how students who use screens for several hours during the day were prone to sleep, physical, and mental health disturbances. The light emitted by phones can also lead to sensory overexcitation that affects the rest cycle, desynchronizing the circadian rhythm [38]. Using phones at night could be harmful as this behavior has been shown to be associated with worsening sleep quality and psychological distress [39].

In our cohort, bad sleep quality, memory difficulties, and reduced performance increased the risk of the onset of the examined symptoms. The relationship between sleep and depressive symptoms and anxiety [40] as well as physical ailments such as headaches [41] and gastrointestinal disorders [42] has already been widely described in the literature as well as the link between epidemics and chronic sleep disturbance [43]. This makes our findings meaningful based on their detection during a pandemic. Previous studies conducted among college students revealed that poor sleep quality and a decreased sleep quantity negatively impacted performance, quality of life, and mental health [44] and had a direct and inverse relationship with anxiety and depression [45] as well as an impact on the onset of panic attacks [46].

Physical activity plays a role in the prevention of depression and a program of physical activity is recommended for individuals with ongoing depression [47]. Our study showed that changes in weight and eating habits had an impact during social isolation on an emergency or protection from the symptoms. Improved eating habits and physical activity have been shown to be protective against sadness/depression as well as headaches and digestive disorders. An increased risk of fear of COVID-19 with this factor was also found. Social isolation impacted the metabolism of students and 35.2% of the sample reported that they increased their weight during quarantine. This trend could be explained by immediate access to food during the quarantine and an increase in the number of snacks during the day [48]. A turnaround was seen in 24.9% of respondents who experienced weight loss. This could stem from concerns about weight gain due to the disruption of sports opportunities (thus reducing the calorie intake) or from increased physical activity at home. A total of 18% of students reported that their eating habits worsened and 25.4% said they had reduced their physical activity. Exposure to stress can alter both the quantity and quality of calorie consumption [49] and stressful events such as the pandemic can lead to the activation of the hypothalamic–pituitary–adrenal neuroendocrine axis (HPA) and increased glucocorticoid synthesis. Glucocorticoids also regulate the accumulation and storage of body fat and can increase appetite, promote food intake, and modify body weight [50]. Activating behaviors of this type can lead to a non-adherence to a varied and healthy diet, leading to gastrointestinal disorders [51], episodes of anxiety and depression [52], and headaches [53]; symptoms also found in our study. The interview revealed alcohol and smoking were associated with headaches, digestive disorders, anxiety and panic, and sadness and depression. This is relevant when thinking about how the young adult population is susceptible to addictive behaviors [54].

The socio-economic questions impacted the psychophysical well-being of the students. Having a low SES level represented a risk factor for the onset of anxiety/panic. It has already been shown that lower social and economic conditions correspond with worse health outcomes and a shorter life expectancy [55]. Our investigation was able to demonstrate how, in cases of social restrictions and adverse conditions, a low perceived SES can represent a risk factor for psychological and physical well-being. This may relate to having fewer resources to help regulate mental well-being such as accessible mental health care.

The results also showed that having symptoms compatible with COVID-19 symptoms (13.0% of the interviewees) or having tested positive for COVID-19 (2.3%) represented risk factors for the emergence of digestive disorders. Having COVID-19-positive or COVID-19-deceased relatives due to the disease represented risk factors for the onset of symptoms such as digestive disorders, anxiety and panic, and sadness and depression. According to recent research, being COVID-19-positive or having symptoms of COVID-19 can trigger four fear domains: (1) fear of/for the body; (2) fear of/for significant others; (3) fear of knowing/not knowing; and (4) fear of taking action/fear of inaction [56]. A fear of the physical consequences caused by the disease, of being a vector of contagion for significant others, and uncertainty about what to do may have impacted the study population, negatively affecting the psychophysical well-being of the students. From the interviews, it emerged that a few lockdown conditions (such as having a private garden, being able to spend hours outdoors, and an increase in the number of cohabitants or rooms in the house) were protective factors against the onset of headaches.

This study can help to advance research to find long-term solutions to what is still an unsolved pandemic and assess the mental health of college students in general. The findings suggest that the COVID-19 pandemic has had an impact on the well-being of young adults. Prevention and early identification with targeted interventions are critical in light of the evidence related to the association between the duration of untreated mental disorders and negative clinical outcomes [57]. Preventive intervention is needed to face the consequences of prolonged social distancing among young adults who continue to study and work in this new environment, along with promoting the adoption and maintenance of healthy behaviors such as undertaking physical exercise, getting enough sleep, and socializing [26]. These findings support the need to implement public health services to reduce healthcare costs. Possible remediations include increasing gender-oriented and specific prevention practices taking into account family commitments. Moreover, interventions should consider psychoeducation about telephone and television use and school education on sleep hygiene and nutrition supported by experienced dieticians. Finally, students should be encouraged to practice tailored physical activity. Habits related to smoking and alcohol consumption should also be limited by the early use of psychoeducation. A functional strategy to address uncertainties about action in the event of an illness should include the use of telemedicine and teletherapy. A wider use of telemedicine and teletherapy can help address mental health conditions [58,59]. Accessible remote medical and mental health care can provide an early diagnosis to minimize the severity of the effects of lockdown and can have direct consequences for a reduction in school dropouts [60].

## 5. Limitations and Further Research

One limitation was that the results may not be generalizable for the entire population. However, the findings were consistent with those of other similar studies targeting university students. In addition, the research involved students primarily from engineering and medicine disciplines and their medical knowledge may have influenced the quality of the answers. Further research can help to determine the generalizability of the results for students of other disciplines. Finally, a few of the measures found through the factor analysis did not show a high reliability (coefficient alpha < 0.7); however, we retained them in the analysis because we were interested in the association between all the different items with the mental health outcomes.

The information was collected after the period of the first lockdown, which could have created recall bias. However, to better understand the nature of the changes experienced, it was considered useful to wait a reasonable time for the symptoms to settle. Furthermore, a real-time evaluation could have created an overestimation bias.

The sample was anonymous, so the same students could not be surveyed again, but it would be informative to compare these findings with a follow-up sample from the university to identify any changes in the conditions or if any chronic elements found in this study persisted.

## 6. Conclusions

Social isolation, in the form of home confinement as a method of combating a COVID-19 infection, had a detrimental effect on the college adult population related to certain lifestyles identified as risk factors. These risk factors have direct health effects and involve the psychosomatic system. Based on our results, practical interventions can be applied or developed to avoid the onset of psychosomatic consequences such as headaches, gastrointestinal disorders, panic and anxiety crises, or episodes of sadness and depression.

## Figures and Tables

**Table 1:** Descriptive statistics of the overall 3533 participants in the survey for the variables included in the factor analysis and in the final models as covariates.

Demographic Information	N (3533)
Age	
Median (Q1, Q3)	22.0 (20.0, 24.0)
Sex	
F	2086 (59.0%)
M	1447 (41.0%)
Socio-economic status (SES)	
Low	474 (13.4%)
Medium	2749 (77.8%)
High	310 (8.8%)
Condition of Social Isolation	
Isolation length	
Median (Q1, Q3)	63.0 (56.0, 76.0)
Access to private garden	
No	1059 (30.0%)
Yes	2474 (70.0%)
Number of cohabitants	
0–1	467 (13.2%)
2–3	2176 (61.6%)
>3	890 (25.2%)
Number of rooms in the house	
<3	251 (7.1%)
3–6	1556 (44.0%)
>6	1726 (48.9%)
Average hours spent outside on a daily basis	
Never	851 (24.1%)
<1 h	1575 (44.6%)
1–3 h	850 (24.1%)
>3 h	257 (7.3%)
Average hours spent using a phone	
≤3 h	1284 (36.3%)
4–6 h	1400 (39.6%)
>6 h	849 (24.0%)
Change in time using a phone	
Same/decreased	872 (24.7%)
Increased	2661 (75.3%)
Average hours spent using a PC	
≤3 h	1071 (30.3%)
4–6 h	1180 (33.4%)
>6 h	1282 (36.3%)
Change in time using a PC	
Same/decreased	837 (23.7%)
Increased	2696 (76.3%)
Average hours spent playing video games	
Never	2097 (59.4%)
<1 h	619 (17.5%)
≥1 h	817 (23.1%)
Change in time playing video games	
Same/decreased	2741 (77.6%)
Increased	792 (22.4%)
Average hours spent watching TV	
<1 h	1606 (45.5%)
1–3 h	1543 (43.7%)
>3 h	384 (10.9%)
Change in time watching TV	
Same/decreased	2411 (68.2%)
Increased	1122 (31.8%)
Lifestyles	
Smoking status during lockdown	
Non-smoker	2594 (73.4%)
Former smoker	175 (5.0%)
Decreased	346 (9.8%)
Same	216 (6.1%)
Increased	202 (5.7%)
Alcohol consumption during lockdown	
Never	655 (18.5%)
Decreased	1700 (48.1%)
Same	962 (27.2%)
Increased	216 (6.1%)
Physical activity	
No	984 (27.9%)
Decreased	896 (25.4%)
Same	441 (12.5%)
Increased	1210 (34.3%)
Usual sleep quality	
Poor	145 (4.1%)
Fairly good	717 (20.3%)
Good	2196 (62.2%)
Excellent	475 (13.4%)
Sleep quality	
Worsened	1468 (41.6%)
Same	1719 (48.7%)
Improved	346 (9.8%)
Change in weight	
Lost weight	878 (24.9%)
No	1411 (39.9%)
Gained weight	1244 (35.2%)
Change in nutrition	
Worsened	663 (18.8%)
No	1851 (52.4%)
Improved	1019 (28.8%)
COVID-19 Information	
Positive for COVID-19	
No/do not know	3450 (97.7%)
Yes	83 (2.3%)
COVID-19 symptoms	
No	3075 (87.0%)
Yes	458 (13.0%)
Relatives infected with COVID-19	
No	3316 (93.9%)
Yes	217 (6.1%)
Relatives with COVID-19 symptoms	
No	3281 (92.9%)
Yes	252 (7.1%)
Relatives died from COVID-19	
No	3464 (98.0%)
Yes	69 (2.0%)
Economic and Financial Situation	
Cohabitant unemployed because of the pandemic	
No	3055 (86.5%)
Yes	478 (13.5%)
Productivity	
Mnemonic difficulties	
No	1863 (52.7%)
Yes	1670 (47.3%)
Performance reduction	
No	1996 (56.5%)
Yes	1537 (43.5%)

**Table 2:** Descriptive statistics of the overall 3533 participants in the survey for the outcome variables.

Outcomes	N (3533)
Digestive disorders	
No	2754 (78.0%)
Yes	779 (22.0%)
Headache	
No	1991 (56.4%)
Yes	1542 (43.6%)
Fear of being infected by SARS-CoV-2	
Low	1081 (30.6%)
Medium	1687 (47.7%)
High	765 (21.7%)
Panic or anxiety crisis	
Low	2250 (63.7%)
Medium	820 (23.2%)
High	463 (13.1%)
Sadness/depression	
Low	930 (26.3%)
Medium	1461 (41.4%)
High	1142 (32.3%)

**Table 3:** Collected variables and latent variables.

Initial Variables	Latent Variables
Average hours spent using a TV; change in time using a TV	TV usage
Average hours spent using a phone; change in time using a phone	Phone usage
Average hours spent using a PC; change in time using a PC	PC usage
Average hours spent playing video games; change in time playing video games	Video games usage
Number of rooms in the house; private garden availability; cohabitants; average hours spent outside	Lockdown conditions
Smoking status during lockdown; alcohol consumption during lockdown	Smoking and alcohol habits
Cohabitants unemployed during lockdown	Unemployed cohabitants during lockdown
Relatives positive for COVID-19; relatives with COVID-19 symptoms; relatives died from COVID-19	Having had relatives positive for COVID-19, with symptoms, or deceased
Having been positive for COVID-19; COVID-19 symptoms	Having been positive for or with COVID-19 symptoms
Physical activity; change in weight; change in nutrition	Nutrition, weight change, and physical activity
Usual sleep quality; lockdown sleep quality; mnemonic difficulties; performance reduction	Sleep quality, mnemonic difficulties, and performance reduction

## Data Availability

Not applicable.

## Supplementary Materials

The following supporting information can be downloaded at: https://www.mdpi.com/article/10.3390/ijerph19148587/s1, Figure S1: Heatmap of the loadings estimated by the factor analysis. Table S1: Descriptive statistics of the overall 3533 participants to the survey.
