# Supplementary material for: COVID-19 Aftermath: Exploring the Mental Health Emergency among Students at a Northern Italian University
Source: Int J Environ Res Public Health. 2022 Jul 14;19(14):8587. doi: 10.3390/ijerph19148587 (PMC9318913; doi:10.3390/ijerph19148587)
Supplement: Supplementary file 1 [file ijerph-19-08587-s001.zip › ijerph-1763019-supplementary.pdf]

Figure S1. Heatmap of the loadings estimated by the factor analysis. Values below 0.31 were set to 0. The factors represented on the y-axis are: F1=having had relatives positive to COVID-19, with symptoms or deceased; F2=videogames usage; F3=sleep quality, mnemonic difficulties and performance reduction; F4=TV usage; F5=lockdown conditions; F6=phone usage; F7=having been positive or with COVID-19 symptoms; F8=PC usage; F9=nutrition, weight change and physical activity; F10=unemployed cohabitants during lockdown; F11=smoking and alcohol habit. The variables displayed on the x-axis are: V1=number of rooms in the house; V2=private garden availability; V3=cohabitants; V4=average hours spent outside; V5=average hours spent using phone; V6=change in time using phone; V7=average hours spent using PC; V8=change in time using PC; V9=average hours spent playing videogames; V10=change in time playing videogames; V11=average hours spent using TV; V12=change in time using TV; V13=smoking status during lockdown; V14=alcohol consumption during lockdown; V15=physical activity; V16=usual sleep quality; V17=lockdown sleep quality; V18=change in weight; V19=change in nutrition; V20=have been positive to COVID-19; V21=COVID-19 symptoms; V22=relatives positive to COVID-19; V23=relatives with COVID-19 symptoms; V24=relatives died for COVID-19; V25=cohabitants unemployed during lockdown; V26=mnemonic difficulties; V27=performance reduction.

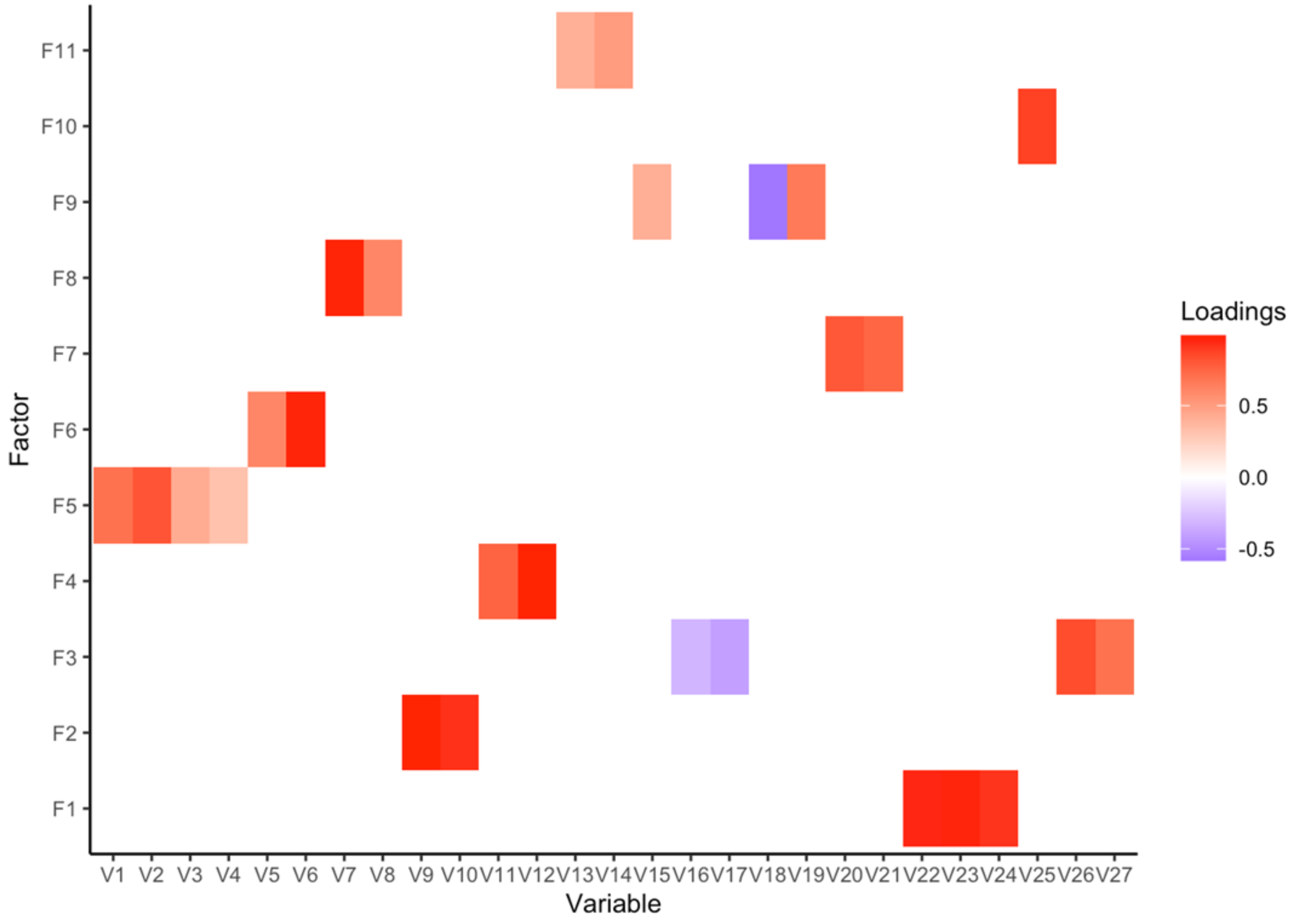

Table S1. Descriptive statistics of the overall 3,533 participants to the survey.

DEMOGRAPHIC INFORMATION	N (3533)
Province	
Altro	536 (15.2%)
Bergamo (BG)	324 (9.2%)
Brescia (BS)	2673 (75.7%)
ACADEMIC AREA	
Medical Science	1389 (39.3%)
Engineering	1096 (31.0%)
Economy	718 (20.3%)
Law	310 (8.8%)
Others	20 (0.6%)
CONDITION OF SOCIAL ISOLATION	
Isolation length categories	
Low	1372 (39.2%)
Medium	1020 (29.2%)
High	1107 (31.6%)
LIFESTYLES	
BMI	
Median (Q1, Q3)	21.7 (19.8, 23.7)
PRODUCTIVITY	
Working in addition to studying	
Never worked before	2290 (64.8%)
No	785 (22.2%)
Yes	458 (13.0%)
Keep studying next semesters	
Yes	3411 (96.5%)
No, for other reasons	89 (2.5%)
No, for pandemic related reasons	33 (0.9%)
Job found during lockdown	
No	2237 (97.9%)
Yes	49 (2.1%)
Cohabitants working previous to lockdown	
0	250 (7.1%)
1	1122 (31.8%)
2	1641 (46.4%)
>=3	520 (14.7%)
Cohabitants kept on working also from home	
0	961 (27.2%)
1	1485 (42.0%)
2	917 (26.0%)
>=3	170 (4.8%)

Prefer online lessons compared to live lessons	
No	2363 (66.9%)
Yes	1170 (33.1%)
